# Fe(II) Spin Crossover/Polymer Hybrid Materials: Investigation of the SCO Behavior via Temperature-Dependent Raman Spectroscopy, Physicochemical Characterization and Migration Release Study

**DOI:** 10.3390/molecules26010201

**Published:** 2021-01-03

**Authors:** Zoi G. Lada, Amaia Soto Beobide, Georgios N. Mathioudakis, George A. Voyiatzis

**Affiliations:** 1 Foundation for Research and Technology-Hellas, Institute of Chemical Engineering Sciences, (FORTH/ICE-HT), Stadiou Str. Platani, GR-265 04 Patras, Greece; asoto@iceht.forth.gr (A.S.B.); mathioy@iceht.forth.gr (G.N.M.); 2 Department of Materials Science, University of Patras, GR-265 00 Rio-Patras, Greece

**Keywords:** spin crossover (SCO) phenomenon, hybrid materials, polymeric composites, variable-temperature micro-Raman spectroscopy, migration release study

## Abstract

Polymeric composites constitute an appealing class of materials with applications in various fields. Spin crossover (SCO) coordination complexes are switchable materials with potential use in data storage and sensors. Their incorporation into polymers can be considered an effective method for their wider practical application. In this study, Fe(II) SCO/polylactic acid hybrid polymeric composites have been prepared by film casting. The mononuclear coordination complex [Fe{N(CN)_2_}_2_(abpt)_2_] was incorporated into polylactic acid. The morphological, structural and thermoanalytical characterization of the composite films were performed via scanning electron microscopy (SEM), attenuated total reflectance (ATR/FTIR), Raman spectroscopy and differential scanning calorimetry (DSC). In addition, the migration release study (MRS) of the SCO compound from the polymeric matrix into the food simulant 50% *v/v* water/ethanol solution was also examined via UV/Vis absorption. Of particular interest was the investigation of the SCO behavior of the coordination complex after its incorporation into the polymer matrix; it was accomplished by temperature-dependent micro-Raman spectroscopy. The described attempt could be considered a preparatory step toward the development of SCO-based temperature sensors integrated into food packaging materials.

## 1. Introduction

Polymeric composites are defined as multiphase materials in which the main phase is a synthetic (bio)polymer incorporating biological/inorganic/organic-derived constituents [1]. They are considered an appealing class of functional materials with applications in various fields, such as construction, electronic devices, energy applications, aerospace and automotive [2,3,4,5,6]. Spin crossover (SCO) compounds are mainly six-coordinate first-row transition metal complexes with d^4^–d^7^ configurations. The SCO phenomenon is referred to the exchange between two well-defined high-spin (HS) and low-spin (LS) states taking place in such coordination complexes. SCO is quite a delicate process and therefore small alterations, such as cooperativity in the solid state through elastic interactions, could tremendously affect its behavior [7,8]. Several studies describe the applications of spin crossover complexes, mainly focused on data storage and sensors [9,10,11,12,13,14]. SCO-based sensors are especially an undoubtedly attractive field. In recent years, the exploitation of SCO-based sensors has evolved at an industrial scale in the form of threshold temperature indicators, irreversible overheat indicators of processes, and so on [15].

The incorporation of SCO complexes into polymers [16,17] has been investigated for several reasons, including convenience for physical characterizations (e.g., photophysical measurements) [18] and competitiveness toward potential technological applications [14]. In addition, the need for the integration and processing of SCO compounds in different shapes and sizes (micro- and nanoscale) has motivated their incorporation into different types of polymer matrices by using spray coating, electrospinning and 3D printing or even by chemical reaction of the SCO system with polymers [19,20]. However, differentiation in the physical and mechanical properties of the SCO material and the polymer matrix after mixing led to the investigation of SCO–matrix interactions based on theoretical modeling and experimental studies [21,22,23]. What is important to investigate, and if possible ensure, is the retention of the functionality of the SCO filler after the process of its incorporation into any composite. In the food packaging sector in particular, it is highly important to certify the effectiveness of the SCO material and its stability after its incorporation into a polymer matrix, mainly as a labeled continuous part of the packaging and not just as a sticker. Moreover, it is important to consider any potential transfer of the material into food media.

**Scheme 1:**
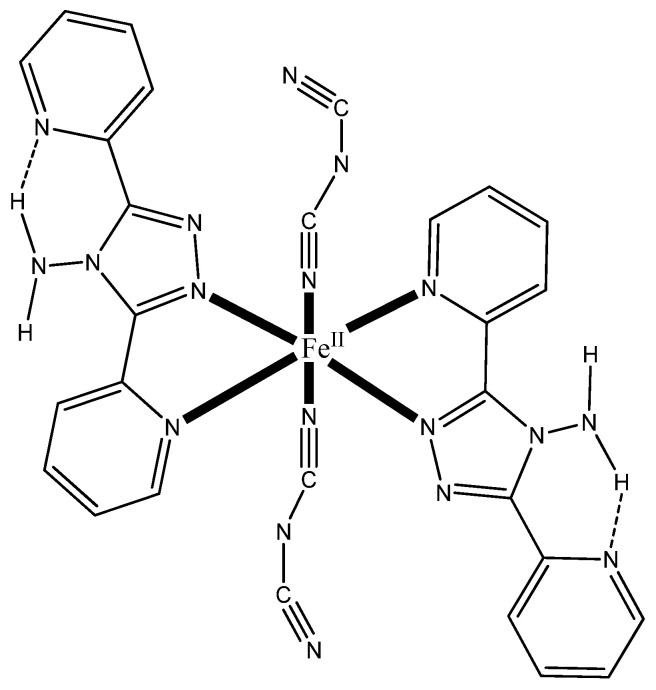
General schematic structure of the [Fe{N(CN)_2_}_2_(abpt)_2_] complex.

The interest of the present study relies on the attempt to integrate a SCO compound (Scheme 1) into a biodegradable polymeric matrix (i.e., polylactic acid-PLA) and subsequently investigate its SCO behavior. In addition, the migration release study of the SCO compound from the polymeric matrix into a food simulant has been also evaluated. These are prerequisites for the future development of SCO-based temperature sensors with more “convenient” characteristics for smart refrigeration/ed food packaging materials (i.e., abrupt transitions at Tc_1_ ≈ 25 °C and Tc_2_ ≈ −50 °C with a hysteresis width ~75 °C). It is worth noting that the SCO behavior after the incorporation of the filler into the polymer has been fulfilled through temperature-dependent confocal Raman microscopy. It is interesting to note that Raman scattered light can be collected via in situ dynamic measurements, e.g., pressure [24,25], temperature [26,27,28,29,30], light-induced [24] and magnetic induction, simultaneously revealing the probed microstructure and spectral features for both the filler and the polymeric matrix. One of the main goals of the present study was to perform a direct correlation of the SCO behavior of the neat complex, investigated previously by in situ Raman, with the corresponding behavior after the incorporation of the SCO complex into a polymer packaging, and simultaneously exploit a technique that not only records the SCO behavior (as magnetic measurements do) but is also able to investigate any alterations at the molecular level of the materials caused by the incorporation process. The morphological, structural and thermoanalytical properties of the polymeric matrix and the filler after incorporation were also examined. The described research activity could be considered a decisive step toward the development and application of SCO-based temperature sensors in food packaging.

## 2. Results and Discussion

### 2.1. Experimental Techniques

**Figure 1:**
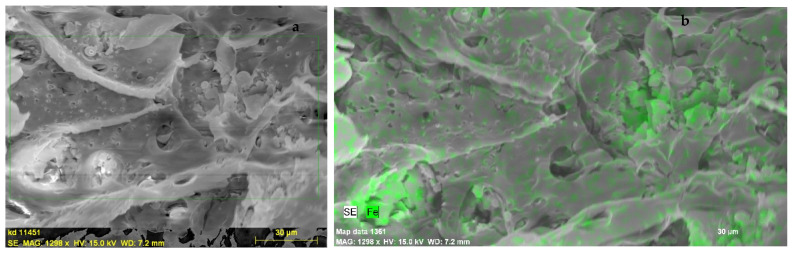
SEM images of a cryogenic cross-section fracture of the hybrid material (**a**) and EDX iron mapping on the same image, with the green color representing the Fe concentration (**b**).

In Figure 1, representative SEM images of the composite (a) after a cryogenic cross-section fracture of the polymeric film, along with the corresponding energy-dispersive X-ray spectroscopy (EDX) analysis (b), are depicted. EDX analysis is an analytical technique used for the elemental analysis or chemical characterization of a sample. The successful distribution of the SCO compound in the PLA polymer matrix, via the green stain of Fe, is clearly revealed. 

### 2.2. Spectroscopic Study

The determination of the main vibrational features for the samples was accomplished via ATR/FTIR and Raman spectroscopy. Raman spectroscopy is a dual-purpose technique, on the one hand highlighting structural features for both the filler (SCO compound) and the matrix (PLA) and on the other hand revealing the SCO behavior of the compound in the composite. The verification that the functionality of the SCO compound remains intact after the incorporation process is crucial and the main task of the present work. 

**Figure 2:**
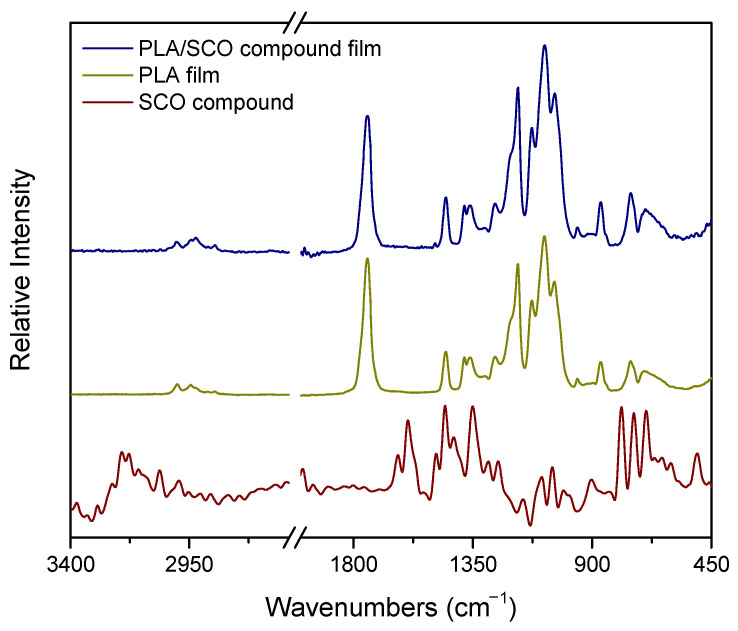
The ATR/FTIR spectra of the PLA/spin crossover (SCO) composite film, the neat PLA and the SCO compound.

The ATR/FTIR spectra of the neat PLA film (middle), the SCO compound (bottom) and the PLA/SCO compound film (top) are shown in Figure 2. The ATR/FTIR spectra of the PLA film before and after the incorporation of the SCO compound are almost identical. In both samples, characteristic vibration absorption bands of PLA are present, such as at 1747 cm^−1^ (-C=O carbonyl stretch), 1456 cm^−1^ (-CH_2_ bend), 1180/1080 cm^−1^ (-C-O- stretch) and 1043 cm^−1^ (-OH bend) [31]. The incorporation of the SCO compound at 0.5% *w/w* into the hybrid material does not appear to contribute to the overall ATR spectrum of the composite. 

**Figure 3:**
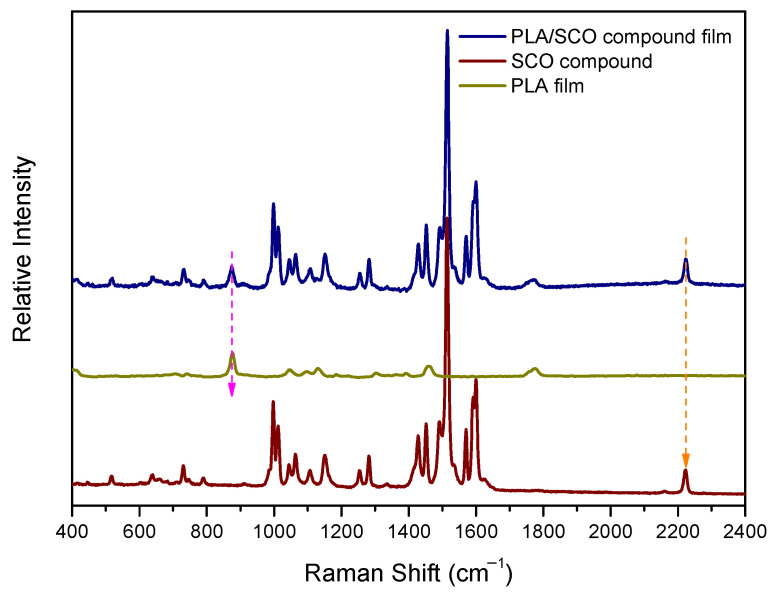
The Raman spectra of the PLA/SCO composite film, the neat PLA and the SCO compound.

The Raman spectra of the neat PLA (middle), the SCO compound (bottom) and the PLA/SCO compound film (top) are shown in Figure 3. The Raman spectrum of the polymeric hybrid material contains scattering spectral contributions of both constituents. A characteristic vibrational feature of each of the two is indicated by dashed arrows. The 875 cm^−1^ peak corresponds to C-COO stretching of PLA [32], and the ~2230 cm^−1^ peak is attributed to stretching vibration of the cyano groups of SCO compound [33,34]. It is interesting to note that the SCO complex exhibits high activity in Raman and very low activity in infrared, always compared to the relevant PLA spectral contributions.

### 2.3. Differential Scanning Calorimetry (DSC) Study

**Figure 4:**
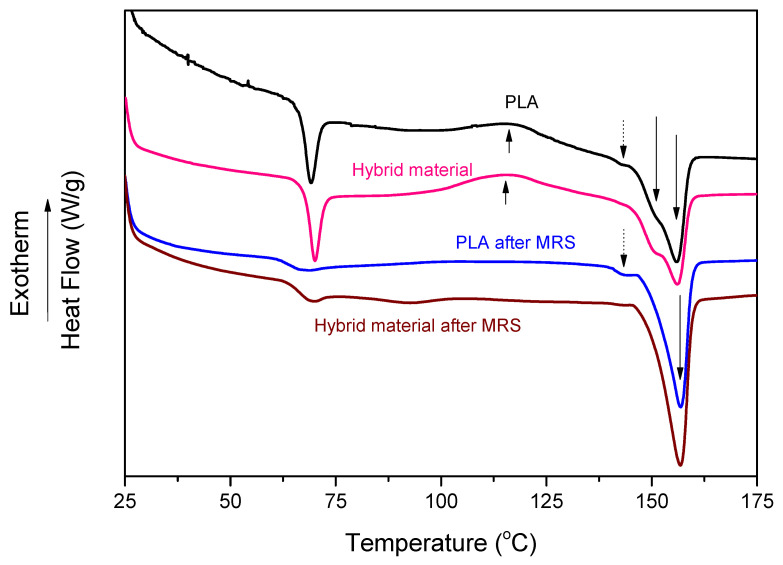
DSC thermograms for PLA and PLA/SCO compound (hybrid material) films before and after migration release study.

Thermal transitions, as well as an evaluation of crystallinity levels of each sample, were determined from first-run DSC scans (Figure 4), referring to the actual state of the polymer specimens. Glass transition temperature (*T_g_*), cold crystallization temperature (*T_cc_*), melting temperature (*T_m_*), crystallization enthalpy (Δ*H_c_*), melting enthalpy (Δ*H_m_*) and crystallinity (*X_c_*) are presented in Table 1. The glass transition temperature (*T_g_*) for the PLA neat film appeared at 66.5 °C, and the addition of the SCO compound had no significant effect on *T_g_*. In both types of samples, the *T_g_* peak exhibits endothermic hysteresis behavior due to overlapping with the coexisting enthalpy relaxation phenomenon that depends on the thermal history of the material [35]. As shown in Figure 4, PLA exhibits three endothermic peaks at 143, 151 and 156 °C. They are mostly related to the melting of certain crystalline phases. PLA can form different crystal phases, including α′-crystals (formed when crystallization occurs below ~120 °C) and α-crystals (>120 °C). The slightly disordered pseudo-orthorhombic PLA, called α’-form, has lower thermal properties than the α-form [36]. In this context, the peak at 151 °C corresponds to the melting of the α′ form that could recrystallize into the α form (α′–α phase transition), and the second peak at 156 °C is related to the melting of the α form [37]. The shoulder endothermic peak at 143 °C in both samples may be tentatively attributed to the presence of PLA oligomers [38] or/and to the existence of a small (~4%) D-isomer units’ content. Interestingly enough, this peak seems to be affected by the migration process and not by the addition of the SCO complex. Regarding crystallinity, it did not appear to be significantly modified from the incorporation of the SCO compound into the polymer matrix, remaining at ~20%. 

The effect of the migration/release study (described below) on the thermal properties of the PLA and the hybrid material was also examined by DSC. The *T_g_* value of the polymeric material was not affected by the migration release study (MRS). On the other hand, the enthalpy relaxation effect was eliminated. The crystallinity of the samples, reflected through the melting endothermic peaks, was significantly affected by the migration process. The melting endothermic peak of the α′ type crystals almost disappeared, while that of the α type crystals increased significantly, suggesting additional, solvent-induced crystallization, by increasing the crystallinity to ~35%. This is consistent with previous similar findings for PLA in contact with ethanolic solvents [39]. 

### 2.4. Migration/Release Study (MRS)

The migration release study is considered important since, in various applications (e.g., smart food packaging materials), potential release of the filler into a dispersion medium (e.g., food) may result in severe consequences. To establish the appropriate experimental conditions for such a study, several directives/regulations have been proposed [40,41,42]. To that end, a release evaluation method using an accredited migration cell and a solution of 50% *v/v* EtOH were used. The specific features associated with the migration cells and the entire migration release study process are described in the Supporting Information. The migration study was performed for both the PLA and the PLA/SCO 0.5% *w/w* composite films. 

**Figure 5:**
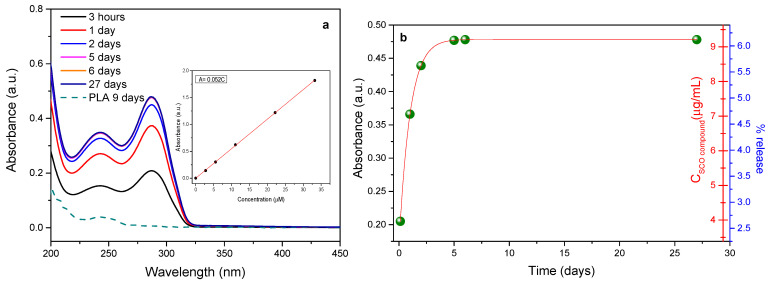
The UV/Vis spectra of the 50% *v/v* EtOH solution in contact with the PLA/SCO compound film using a migration cell (**a**) and the subsequent concentration and percentage of the SCO compound released into the 50% EtOH solution (**b**) as a function of time. In the inset, the calibration curve plot absorbance versus the concentration extracted from the spectra is shown.

Figure 5a shows the UV/Vis absorption spectra of the solution in contact with the PLA/SCO compound film bound to the migration cell as a function of time, while Figure 5b indicates the resulting concentration and percentage of the SCO compound released in the 50% EtOH solution for the various times. As noticed within the first hours, there was a clear increase in the release. This reached a plateau value after five days, with a total release being about 6% of the total amount of SCO compound in the film. This behavior could be most properly described as a leaching rather than a migration process. Most probably, part of the SCO material that did not fully integrate into the PLA matrix but remained on its surface was easily released into the simulated solution during the early stage of migration. This is further supported by a second migration/release study, which was performed by using the sample used in the first migration study. As it was revealed, no further release was noticed on this second-run migration study (Figure S3). The relatively low overall release of the additive may be attributed to the high activation energy values required for its diffusion by the PLA/SCO nanocomposites. The existence of corresponding high energy values has also been mentioned in other PLA-based nanocomposites [43,44]. The release of pure PLA was also considered. An absorption band at ~245 nm was present after nine days in the neat PLA film. This band remained stable at its maximum absorption even after the 27 days of the total duration of the migration process, in line with the stability already shown by the PLA in similar conditions to the present study [45]. Even if the categorization of the SCO substance as acceptable or not concerning its release limit is not profound, and an independent research activity referring to the toxicity limits of this system is required, according to EU regulations, there is a general limit in which the upper acceptable limit of substances in contact with food is 10 mg of substance/1 dm^2^ of packaging material. Based on this limit, in our case, even if most of the amount of the SCO compound was released into food simulant (which is not the case and only 6% is released), we will fairly cover this requirement of the EU [46].

### 2.5. The SCO Behavior of [Fe(abpt)_2_{N(CN)_2_}_2_] after the Incorporation in the Polymer Matrix

**Figure 6:**
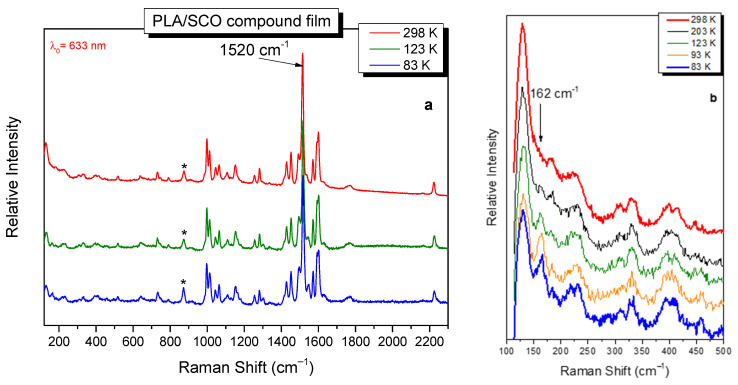
The Raman spectra of PLA/SCO compound film for various temperatures (**a**) and the low-frequency region of the Raman spectra after normalization (**b**). A noticeable difference in the spectra is the appearance of the band at 162 cm^−1^ at the low temperatures examined. The characteristic band of PLA at ~870 cm^−1^ is indicated with an asterisk in the Raman spectra.

As already mentioned, the main aim of this work is to examine/verify the SCO ability of the material after its incorporation into the polymer matrix. For that reason, Raman spectra of the SCO compound and the polymeric hybrid material were compared. In Figure 6a, representative Raman spectra at selected temperatures for the PLA/SCO compound film are shown. In Figure 6b, the low-frequency region of the relevant spectra after normalization (based on the “not sensitive to SCO” band at 1520 cm^−1^) is also presented [26]. 

Differences in the Raman spectra were shown decreasing the temperature. The most noticeable is the appearance of the band at 162 cm^−1^, which is attributed to Fe-N stretching vibrations of the SCO complex in the LS state. This band was previously selected for monitoring the SCO process via Raman in the initial coordination complex [26]. It is noted that the corresponding Fe-N of the HS state is anticipated to be located at the ultra-low-frequency region at ~50 cm^−1^.

**Figure 7:**
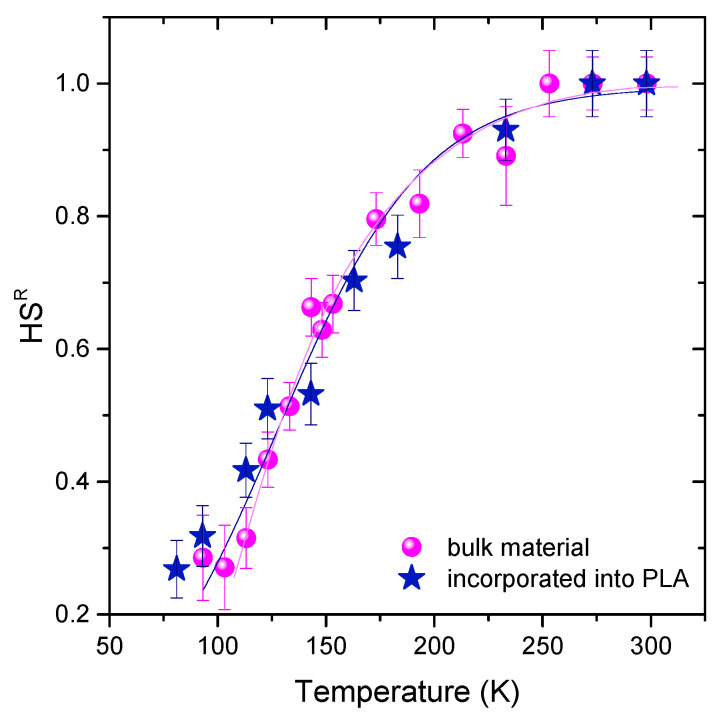
Temperature dependence of the high-spin (HS) population extracted from the Raman spectra of the hybrid material. The methodology used for calculating the HS populations was based on our earlier study, and the corresponding previously described data of the bulk SCO compound are also presented for comparison [26]. Reprinted with permission from Zoi G. Lada, Konstantinos S. Andrikopoulos, Athanassios Chrissanthopoulos, Spyros P. Perlepes, and George A. Voyiatzis *Inorganic Chemistry* **2019** 58 (8), 5183-5195). Copyright (2020) American Chemical Society.

In order to ensure that the effectiveness of the SCO functionality remained during the incorporation process, the temperature-dependent Raman spectra of the polymer hybrid material were compared with the corresponding neat SCO compound; they were found to be closely comparable. Figure 7 depicts the temperature dependence of the HS population extracted from the Raman spectra of the hybrid material. The total SCO behavior of the compound (curve motif and T_1/2_ value) did not seem to be affected after the incorporation of the SCO compound into the matrix. This stability of the SCO functionality after incorporation is considered an important finding for the perspective of practical implementation of SCO compounds.

It is important to highlight that this is the first attempt in which a SCO compound is incorporated into a polylactic acid (PLA) matrix. Moreover, the effect of filler incorporation on the SCO behavior and the physical properties of the polymeric matrix was examined and accompanied by the simultaneous migration release study of SCO/polymer hybrid materials. The result of this study could be a prelude to the practical application of SCO materials, obtaining a broad assessment of the properties of SCO-based hybrid composites.

## 3. Experimental Section

### 3.1. Materials and Procedures

The synthesis of the nanosized (~20 nm) SCO compound [Fe(abpt)_2_{N(CN)_2_}_2_] (polymorph **B**) and the detailed SCO behavior of the nanoparticles (investigated through temperature-dependent Raman spectroscopy) has been previously described [26]. The thermoplastic polylactic acid (PLA) used in the present study was of commercial grade (Ingeo PLA 3052D by Nature Works LLC, allegedly containing ~4% D-lactide isomer) kindly supplied by ex-ARGO S.A. PLA is a biodegradable polymer widely used in recent decades in various applications especially as packaging material for consumer goods. It should be noted that commercial PLA grades typically comprise the L-isomer, but a small percentage of D-isomer units are also present in chains that could affect the PLA, something that is often neglected in the determination of the thermodynamic properties of PLA [47]. The preparation method selected for the Fe(II) Spin Crossover/polymer hybrid material was film casting, considered a nondestructive method for the incorporation of a compound into a biopolymer matrix. Prior to composite preparation, the solubility of both the polymer matrix and the SCO compound was examined in a variety of solvents. The solvent selected was CH_2_Cl_2_ (dichloromethane), with a SCO compound/PLA ratio in the composite of 0.5% *w/w* (details on the film preparation are presented in the Supporting Information). The filler was well dispersed in the polymer matrix, resulting in satisfactory homogeneity of the obtained film. The color of the film at RT was pale pink (HS form of the SCO complex), and after immersing in liquid N_2_ (T = 77 K), the color of the film turned purple (LS form of the SCO complex). Alterations in the structural bond distances occurring during the spin crossover transition led to color variation between the two states [26]. 

### 3.2. Experimental Techniques

The morphology of the polymeric composites was determined by scanning electron microscopy (SEM). A Zeiss ZUPRA 35 VP-FEG instrument, operating at 5−20 keV, was used. The system was equipped with EDS (Bruker GmbH, Quanta 200, MA, USA) and BSE (K E Developments, Ltd., Cambridge, UK), which enabled elemental analyses.

For the collection of the Raman spectra, the T64000 Horiba Jobin Yvon micro-Raman setup was used. The 632.8 nm wavelength emitted from the HeNe laser source (Optronics Technologies S.A., Model HLA-20P, 20 mW, Moschato, Greece) was used for the excitation of the samples after being focused by a 50× microscope objective (Olympus, NA 0.55, Tokyo, Japan). The collected backscattered radiation was directed to the monochromator (single configuration) after passing through an appropriate edge filter (LP02-633RU-25, laser2000, UK, Ltd., Huntingdon, Cambridgeshire, UK) and detected by a LN-cooled CCD detector (Symphony^®^ II, Horiba Scientific, Kyoto, Japan). Instrumental calibration was performed via the standard 520.5 cm^−1^ Raman peak position of a Si wafer. The spectral resolution was 5 cm^−1^. Temperature-dependent spectra were acquired with the use of a cooling stage (temperature-controlled stage, THMS600/720, LINKAM), which enabled temperature stabilization better than 0.5 K throughout the temperature range from the liquid nitrogen up to ambient. The ratio of the integrated intensities of the characteristic HS and LS form bands was determined by deconvolution performed in triplicate for different spots on the sample for statistical reasons. 

Attenuated total reflectance (ATR) spectra were recorded on a Bruker Optics Alpha-P Diamond ATR spectrometer of Bruker Optics GmbH.

Differential scanning calorimetric (DSC) measurements were made using a TA instruments Q100 thermal analyzer at a heating rate of 10 °C/min. The DSC measurements were performed 27 days after film preparation to compare with those used in the migration release study (MRS), which also lasted 27 days. Thermal transitions as well as an evaluation of crystallinity levels of samples were determined from the first DSC runs. The glass transition temperatures (*T_g_*) of samples were collected from the midpoint of the stepwise specific heat increment. The degree of crystallinity (*X_c_*) of the samples was determined according to Equation (1):

(1) $$X_{C}=\;\frac{\Delta H_{m}-\Delta H_{c}}{\left(1-\omega \right)\Delta H_{m}^{0}}\;\times \;100\%\;$$

where Δ*H_m_* and Δ*H_c_* are the enthalpies of melting and cold crystallization, respectively, and *ω* is the SCO compound weight fraction. Δ*H*^0^*_m_* is the melting enthalpy of 100% crystalline PLA given as 93.7 J/g [48].

The migration study was performed using appropriate stainless steel migration cell systems. The specified cells (Figure S1) were purchased from LABC-Labortechnik (http://www.labc.de/). The cells were placed in a shaking incubator (Wise Cube) at a stable temperature (40 °C) for a period of 27 days [46]. According to EU regulations, the time required to consider the migration release of packaging materials in food simulators is 10 days. In the present work, however, the migration release study was monitored for 27 days to allow a clear estimate of the total migration of the hybrid material in a longer period of time that would cover possible degradation of the biopolymer itself or/and allow any natural aging to reach an equilibrium over slow rearrangements [49]. Details on the migration release study, monitored through UV/Vis absorption recorded on a Shimadzu UV-1900 spectrometer, are given in the Supporting Information. The released medium utilized was the type D1 food simulant in a 50% *v/v* ethanol/water solution (suitable for preserved fruits, vegetables and meat). Τhe released concentration of the SCO compound was quantitatively determined by correlating the recorded band intensity of the characteristic adsorption peak at 275 nm to the calibration curve constructed with UV/Vis spectra of known concentrations of the SCO compound in 50% EtOH solutions (Figure S2).

## 4. Conclusions

The main goal of the present study was the development of a SCO–polymer hybrid material and the subsequent investigation of the effect on both the filler (SCO behavior of the compound) and the matrix’s (polymer) properties after the incorporation of the SCO compound into the polymer matrix. As revealed from the temperature-dependent Raman spectroscopy, the SCO behavior of the compound remained unaltered after its incorporation into the polymer matrix. In addition, the potential migration of the SCO material into the solution was also examined. The contact of the samples with the ethanol solution for migration/release study appeared to mainly affect the crystallinity of the samples, which is attributed to solvent-induced crystallization. In this context, an exponential release observed in just the first two days is attributed to a leaching rather than a migration process. No further release was subsequently recorded, with the plateau of the release corresponding to ~6% of the total amount of SCO compound incorporated into the PLA polymer. The importance of this study is emphasized by the lack of relevant studies on SCO/polymer hybrid systems and the importance of being able to implement SCO systems in various applications. 

## Figures and Tables

**Table 1:** Thermal properties of neat PLA and hybrid PLA/SCO compound films based on DSC results.

Sample (Hybrid Material)	T_g_ (°C)	T_cc_ (°C)	T_m_ (°C)	Crystallinity %
PLA	66.5	116.5	143 (sh), 151, 156	21.5
PLA after migration	65		143 (sh), 157	37
Hybrid material	67	114.70	151, 156	19
Hybrid material after migration	67		157	32

## Supplementary Materials

The supplementary materials are available. Figure S1: (a) The components of the migration cells. Cells of three different geometrical characteristics; the cell possessing 30 mm diameter was used for the migration experiments. (b) A typical assembly of the cell that is ready to be placed into the incubating chamber, Figure S2: The UV-Vis spectra of the [Fe(abpt)_2_{N(CN)_2_}_2_] coordination complex in 50% EtOH for various concentrations (a) and the subsequent calibration curve based on the maximum value of the absorbance at 287 nm (b), Figure S3: The UV-Vis spectra of the 50% *v*/*v* EtOH solution in contact with the PLA/SCO compound film for a second migration/release study as a function of time; the same scale as the one used in the first migration study (Figure 5) has been used for direct comparison. In the inset is shown in a greater detail the UV-Vis spectra.

## Sample Availability

Samples of the compounds are not available from the authors.
