# Supplementary material for: Fe(II) Spin Crossover/Polymer Hybrid Materials: Investigation of the SCO Behavior via Temperature-Dependent Raman Spectroscopy, Physicochemical Characterization and Migration Release Study
Source: Molecules. 2021 Jan 3;26(1):201. doi: 10.3390/molecules26010201 (PMC7796439; doi:10.3390/molecules26010201)
Supplement: Supplementary file 1 [file molecules-26-00201-s001.pdf]

Fe(II) Spin Crossover/Polymer Hybrid Materials: Investigation of the SCO Behavior *via* Temperature Dependent Raman Spectroscopy, Physicochemical Characterization and Migration Release Study

Zoi G. Lada [1,*], Amaia Soto Beobide [1], Georgios N. Mathioudakis [1,2] and George A. Voyiatzis [1,*]

[1] Foundation for Research and Technology-Hellas, Institute of Chemical Engineering Sciences, (FORTH/ICE-HT), Stadiou Str. Platani, 265 04 Patras, Greece

[2] Department of Materials Science, University of Patras, GR-265 00 Rio-Patras, Greece

* Correspondence: Zoi G. Lada, zoilada@iceht.forth.gr, Tel.: +30 2610965241; George A. Voyiatzis, gvog@iceht.forth.gr, +302610965253.

Hybrid SCO/PLA Composite Preparation Procedure

Before the film preparation, the solubility for both, the polymer matrix and the SCO compound, were examined in a variety of solvents. The selected solvent for the dissolution of PLA and the SCO compound was CH_2Cl_2 (dichloromethane). The PLA membrane containing the SCO compound in a composition 0.5% w/w was prepared by the solution casting technique. The general film preparation procedure followed in our case involves:

- ✓ Separated dissolution of the polymer matrix and the SCO compound in a suitable solvent under magnetic stirring.
- ✓ Addition of the SCO compound solution in the polymer matrix solution under magnetic stirring. The mixture was left under stirring for ~10 min in order to ensure that a homogenous solution was resulted.
- ✓ Deposition of the mixture in a Petri dish which was left at RT for 24 h until solvent evaporation.
- ✓ The film prepared was then put in a vacuum oven equipped with an oil pump (T= 40 ^{o}C) in order to make sure that all the amount of solvent was evaporated.
- ✓ The films had a thickness of 100-120 μm.

Migration Release Study

The migration study was performed by using stainless steel migration cell systems appropriately used. The specified cells (Figure S1) were purchased from LABC-Labortechnik (http://www.labc.de/). The cells were placed in the shaking incubator at stable temperature (40 ^{o}C) for a period of 27 days. At several time intervals, 2 mL aliquots were removed and UV-Vis absorption spectra were measured in order to be checked with respect to migrating species and the aliquots were subsequently returned back in the cell after the measurements. The required calculations (calibration curves of the fillers) for the accurate quantitative determination of the migrated quantity of each filler were made before the measurements. The duration of the migration study was 27 days and the technique for monitoring the migration study was UV-Vis spectroscopy.

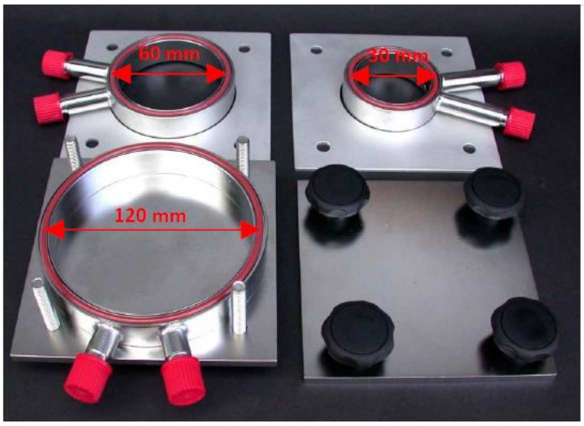

(a)

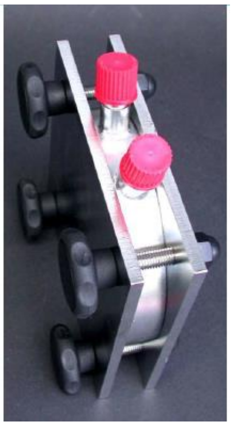

(b)

Figure S1. (a) The components of the migration cells. Cells of three different geometrical characteristics; the cell possessing 30 mm diameter was used for the migration experiments. (b) A typical assembly of the cell that is ready to be placed into the incubating chamber.

In Figure S2 the UV-Vis spectra of solutions of the SCO compound in 50% EtOH (left) and the subsequent calibration curve (right) are shown. The calibration curve was based on the maximum value of the absorbance of the complex at the 287 nm.

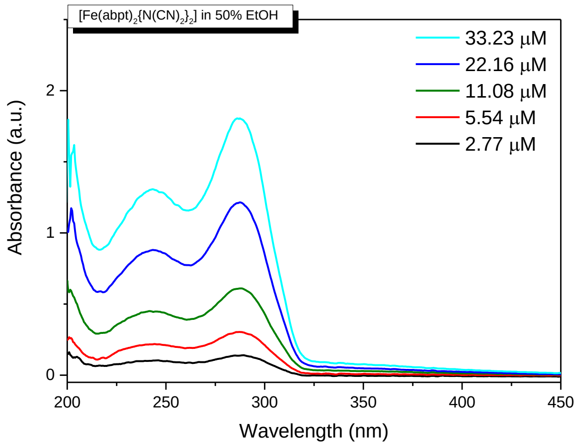

(a)

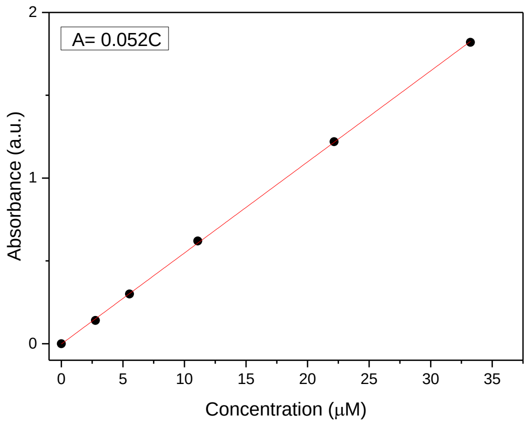

(b)

Figure S2. The UV-Vis spectra of the $[Fe(abpt)_2\{N(CN)_2\}_2]$ coordination complex in 50% EtOH for various concentrations (a) and the subsequent calibration curve based on the maximum value of the absorbance at 287 nm (b).

A second migration/release study with the sample used in the first study was performed. After the conclusion of the migration/release study performed and presented in figure 5, the sample was removed from the migration cell and placed in a new migration cell with fresh solution of 50% v/v EtOH. The second migration/release study lasted 6 days. The results from this second migration/release study are depicted in Figure S3. We can notice that the release is negligible, confirming leaching during the first hours/days rather than a migration process. As an alternative, the composite should be washed prior to its potential commercial use.

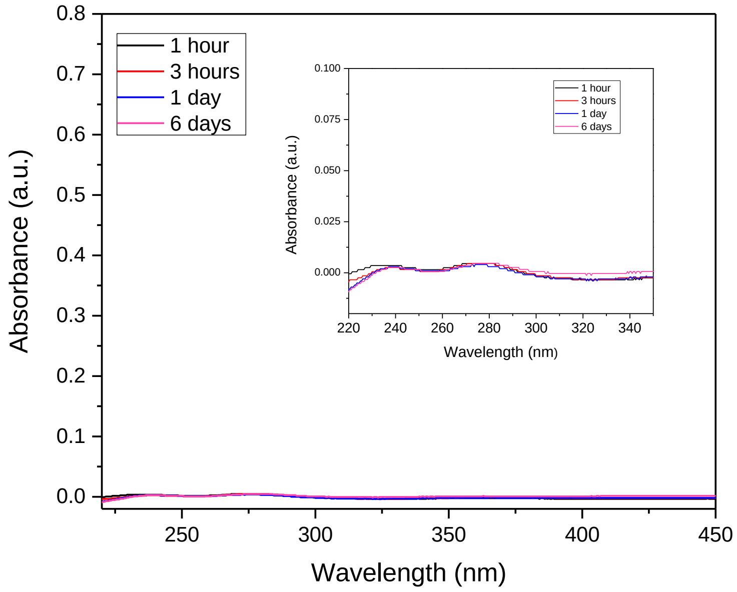

Figure S3. The UV-Vis spectra of the 50% v/v EtOH solution in contact with the PLA/SCO compound film for a second migration/release study as a function of time; the same scale as the one used in the first migration study (Figure 5) has been used for direct comparison. In the inset is shown in a greater detail the UV-Vis spectra.
